# Using EMPOWER in daily life: a qualitative investigation of implementation experiences

**DOI:** 10.1186/s12888-023-05096-x

**Published:** 2023-08-17

**Authors:** Stephanie Allan, Sara Beedie, Hamish J. McLeod, John Farhall, John Gleeson, Simon Bradstreet, Emma Morton, Imogen Bell, Alison Wilson-Kay, Helen Whitehill, Claire Matrunola, David Thomson, Andrea Clark, Andrew Gumley

**Affiliations:** 1 University of Glasgow, Glasgow, UK; 2 NHS Greater Glasgow & Clyde, Glasgow, UK; 3 La Trobe University, Melbourne, Australia; 4 Australian Catholic University, Melbourne, Australia; 5 University of British Columbia, Vancouver, Canada; 6 Orygen Centre of Youth Mental Health, Melbourne, Australia

**Keywords:** Schizophrenia, Implementation science, Mhealth

## Abstract

**Background:**

Digital self-management tools blended with clinical triage and peer support have the potential to improve access to early warning signs (EWS) based relapse prevention in schizophrenia care. However, the implementation of digital interventions in psychosis can be poor. Traditionally, research focused on understanding how people implement interventions has focused on the perspectives of mental health staff. Digital interventions are becoming more commonly used by patients within the context of daily life, which means there is a need to understand implementation from the perspectives of patients and carers.

**Methods:**

Semi-structured one-on-one interviews with 16 patients who had access to the EMPOWER digital self-management intervention during their participation in a feasibility trial, six mental health staff members who supported the patients and were enrolled in the trial, and one carer participant. Interviews focused on understanding implementation, including barriers and facilitators. Data were coded using thematic analysis.

**Results:**

The intervention was well implemented, and EMPOWER was typically perceived positively by patients, mental health staff and the carer we spoke to. However, some patients reported negative views and reported ideas for intervention improvement. Patients reported valuing that the app afforded them access to things like information or increased social contact from peer support workers that went above and beyond that offered in routine care. Patients seemed motivated to continue implementing EMPOWER in daily life when they perceived it was creating positive change to their wellbeing, but seemed less motivated if this did not occur. Mental health staff and carer views suggest they developed increased confidence patients could self-manage and valued using the fact that people they support were using the EMPOWER intervention to open up conversations about self-management and wellbeing.

**Conclusions:**

The findings from this study suggest peer worker supported digital self-management like EMPOWER has the potential to be implemented. Further evaluations of these interventions are warranted, and conducting qualitative research on the feasibility gives insight into implementation barriers and facilitators, improving the likelihood of interventions being usable. In particular, the views of patients who demonstrated low usage levels would be valuable.

**Supplementary Information:**

The online version contains supplementary material available at 10.1186/s12888-023-05096-x.

## Background

Almost half of all people with schizophrenia will relapse within five years post-diagnosis [1]. Because standard treatment with antipsychotics does not entirely prevent relapse [2] adjunctive psychosocial approaches are recommended [3]. A common psychosocial approach to relapse prevention is to detect and respond to early warning signs (EWS) [4, 5], but the demand for this type of psychosocial support typically outstrips mental health service capacity [6]. Digital interventions provide one way to upscale access to psychosocial interventions and offer more autonomous service engagement options for people diagnosed with schizophrenia [7]. However, digital interventions for psychosis can be poorly implemented [8] which means there is a need to develop and evaluate interventions which are a good fit within clinical services and by patients in their everyday life.

Within clinical services, EWS-based relapse prevention relies on patients, mental health staff and carers monitoring for EWS and reacting promptly to prevent a relapse event which appears to reduce relapse rates [5]. However, this approach comes with the risk of false positives where mental health staff may overreact to typical fluctuation, which may alarm patients and their carers, leading to them avoiding reporting EWS because they fear the response of mental health services [9]. Due to this uncertainty, current EWS-based relapse prevention [10] is best described as a complex social process where mental health staff, carers and patients weigh up the risks and benefits of responding to EWS.

The Early Signs Monitoring to Prevent Relapse in Psychosis and Promote Well-Being, Engagement, and Recovery (EMPOWER) intervention [11] was designed with the problems of traditional EWS based relapse prevention in mind to offer patients safety and self-efficacy. This was achieved through smartphone technology which was designed to foster awareness of symptoms and affective experiences over time. Patients were invited to self-monitor for up to a year; the app was responsive to user input and provided tailored self-management information. For example, if user input signalled a person was struggling with voices, they were given a message about voice content. Patients could view charts to see their own data and could choose to share this data with their mental health staff or carers. The evaluation of the EXPRESS and FOCUS studies which were similar to EMPOWER as they offered self-monitoring, suggests that people with psychosis find frequent self-monitoring acceptable for up to six months [12, 13], but less is known about how patients might experience self-monitoring via an app in the longer term. Additionally, as mental health staff and carers have a crucial role in EWS management – it is important to find out how they respond to people who they support through self-monitoring and how this might fit into daily life. This is particularly important because it is well recognised mental health staff are constrained by a lack of time and are unlikely to support an intervention which increases staff burden [14].

Human contact is important for active engagement in digital interventions for psychosis [15]. EMPOWER offered two types of human contact. Peer support workers with their own experience of mental health problems have been identified as important in supporting patients in learning about and implementing relapse prevention plans [16]. EMPOWER offered fortnightly appointments with peer support workers to discuss self-management and offer support in using the intervention. If a patient inputted data which suggested a marked change, they would also be contacted by a study team member. In recognition that fluctuations are likely in the context of recurring psychosis – the data gathered during self-monitoring was subject to clinical triage, which enabled timely human support and shared decision making from an experienced mental health nurse or clinical psychologist if they deemed it necessary. More information on clinical triaging can be found in the main study outcomes paper [11].

The primary trial outcomes indicated that overall EMPOWER was feasible, acceptable and safe. This was indicated by high levels of recruitment and retention, the intervention meeting its a-priori feasibility criteria for intervention adherence and low levels of adverse events. Due to this result, there is need to understand how and why the intervention was implemented in the context of this trial. Implementation behaviours describe what people do when exposed to a new intervention, and understanding implementation behaviours require consideration of context and influences on behaviour (including subjective experiences) [17]. It is possible to learn about implementation barriers for psychosocial interventions by studying poorly implemented interventions [18], but relying on retrospective data might miss key information about relevant factors that emerge during the implementation process [19, 20]. Furthermore, conducting implementation research during feasibility trials means that strategies to overcome problems can be identified before progression to a full-scale trial [21].

Implementation research has historically focused on the experiences of healthcare staff [22], possibly part of a general pattern within healthcare research where the views of patients and carers are devalued [23]. However, this is changing and current guidance is that researchers should access the experiences of all relevant stakeholders to understand implementation experiences [24]. This is especially pertinent for interventions used independently by patients.

Process evaluations are useful for understanding implementation behaviours and what underpins them because these studies investigate the different components of a complex intervention, how it is delivered, and what happens when people interact with an intervention. Process evaluations conducted in feasibility trials can improve the validity and interpretation of outcomes, help refine the intervention, and provide necessary information to help inform upscaling decisions or outline the need for intervention refinement [21]. Therefore, we aimed to explore implementation behaviours that occurred in the EMPOWER feasibility trial from the point of view of carers, patients and mental health staff through interviews. Our findings were intended to aid our understanding of what underpinned implementation behaviours to help guide decisions about upscaling into a full-scale clinical trial of a peer support worker supported digital self-management tool for psychosis. As we did not know what would underpin implementation behaviours in advance and we were interested in understanding implementation behaviours from the participant’s point of view, it was decided to use inductive methods.

## Methods

### Setting

This qualitative study occurred during the conduct of the EMPOWER trial while participants were randomised to have access to the intervention. The protocol and main trial outcomes have been published [11, 25]. The study was conducted in Australia and Scotland with 73 people diagnosed with schizophrenia spectrum conditions who had relapsed within the past two years. The participants were recruited from community mental health teams were randomised to receive either the intervention (n = 42) or treatment as usual (n = 31). In total, 30 of the patients randomised were based at the UK site and 12 patients were based in Australia. In total, seven carers consented to taking part in the study and 22 mental health staff enrolled.

### Participants

This qualitative study was embedded within the EMPOWER trial and received ethical approval from West of Scotland Research Ethics Service (16/WS/0225) and Melbourne Health Human Research Ethics Committee (HREC/15/MH/334). All participants provided their informed and written consent before participating in the process evaluation interviews. Patients were eligible if they were randomised to receive the EMPOWER intervention and had not withdrawn informed consent.

### Intervention

EMPOWER [11] was a feasibility cluster randomised controlled trial of a digital EWS self-monitoring app blended with peer support and clinical triage for people diagnosed with schizophrenia spectrum disorders. As described in the trial protocol ([11], p.8): “EMPOWER was developed as a flexible user-led tool to (1) daily monitor the ebb and flow of changes in [patient] well-being which incorporated, (2) personalized EWS items, (3) enabled the delivery of EMPOWER (self-management) messages directly to patients and, (4) provided a mobile phone user interface to enable patients to review their own data and keep a diary of their experiences.” Three peer support workers (one in Australia and two in Glasgow) were employed to help set up the app for participants and provide regular fortnightly telephone support. If participants were digitally excluded [26] and did not already own a smartphone, they were supplied with a phone and data. Participants had access to EMPOWER for up to 12 months.

### Procedures

Prior to onset of interviews, SA attended weekly team meetings to understand the conduct of the trial and updated SBe on what was happening so that the semi-independent process evaluation team were aware of the context of the trial. EMPOWER participants were invited to take part in interviews to understand their experiences. Service user participants were purposively sampled with reference to gender and intervention engagement. This was chosen because at the two-month point after participants were randomised, concern was raised at team meetings that men were engaging less with the app and peer support, and we felt it would be beneficial to explore these differences qualitatively. Linked to this, it was decided it would be important to speak to participants with differing levels of intervention usage.

Participants were first approached by members of the trial team that they had contact with such as the trial managers, peer support workers, clinical triage staff and research assistants. All trial staff were briefed on the study and asked patient participants if they would like to find out more about the study and be contacted by SA or SBe. The study was described as an opportunity to speak to someone about their experiences who was independently evaluating the EMPOWER intervention. All recruitment occurred via trial staff. The staff working on the trial were aware of the wellbeing of participants and sometimes expressed that they felt an interview would be burdensome for participants experiencing high levels of distress which minimised the number of people who could be contacted for an interview.

Nonetheless, staff were encouraged to identify people whom they felt were using the app at low levels. During team meetings, it was agreed with trial staff that speaking to 50% of patients still enrolled in the study would generate a variety of viewpoints on the intervention and give adequate information power [27] to address the study aims without overburdening patient participants. When being interviewed, patient participants were asked if they consented to mental health staff or carers being interviewed – while this limited the number of mental health staff and carers who could be spoken to, this ensured a patient centred approach. When contacted by SA and SBe, mental health staff and carers were informed the interview would seek to understand their views.

The two process evaluation interviewers (SA and SBe) conducted all interviews. They were female, experienced in qualitative methods as part of doctoral research training and had no existing relationships with participants. All participants gave written and informed consent. One-on-One interviews with UK-based participants were conducted face-to-face with patients (n = 12) and a carer (n = 1) and interviews with UK-based staff (n = 5) were conducted by SBe as part of her doctoral training in clinical psychology. Interviews with Australian patient participants (n = 4) and a mental health staff member (n = 1) were conducted by SA over the telephone. Interviews with UK carer and patient participants were conducted in people’s homes. Mental health staff were interviewed in their place of work. SA and SBe took notes during interviews. Everyone was interviewed during trial participation to minimise retrospective recall biases or loss of recall detail. Interviews were conducted between 14/11/2018 and 18/06/2019, and all of these dates were prior to participants no longer having access to the intervention at the end of June 2019. All interviews were audio-recorded and then transcribed verbatim, participant details were anonymised, and people referred to by a pseudonym. Transcripts were not returned to participants due to time limitations.

Further information on the development of the pilot tested interview schedules (and actual copies of the schedules) can be seen in the pre-published protocol [28]. First, however, we present a summary here:

The service user interview schedule covered:

- Experiences of using EMPOWER.
- Experiences of implementing EMPOWER in the context of daily life.
- Experiences of intervention components.
- Experiences of data sharing.
- Suggestions for improvement.

The mental health staff interview schedule covered:

- Experiences of supporting a patient taking part in EMPOWER.
- Experiences of interacting with EMPOWER in clinical practice.
- Experiences of data sharing.
- Suggestions for improvement.

The carer interview schedule covered:

- Experiences of supporting a patient taking part in EMPOWER.
- Experiences of data sharing.
- Suggestions for improvement.

### Analysis

All transcripts were analysed using inductive thematic analysis [29] by SA as the primary analysist. This analysis has six steps: (1) becoming familiar with the data, (2) generating initial codes – where descriptive codes were initially constructed, (3) developing initial themes, (4) reviewing themes, (5) defining themes, and (6) writing the report. Thematic analysis was guided throughout by the research aims (to understand implementation) and was an iterative process that involved comparing and contrasting codes both between and interviews to construct themes. As highlighted by Byrne [30], the boundary between (5) “defining themes” and (6) “writing the report” in thematic analysis can be blurry, and both steps 5 and 6 are an active part of the analytical process. In the case of this thematic analysis, stage 6 involved theoretically framing the thematic analysis as there was recognition that an overarching theme is what the intervention afforded (and did not afford) participants. Affordances, first theorised by Gibson [31] describe the process by which people perceive possibilities for action from an object in their environment [32]. The theoretical framework of affordances, which as applied to EMPOWER, is discussed further within the results section was not identified a-priori and came into the analysis during (6) “writing the report”. During stage six, the analytic procedure may be considered to have become deductive in nature due to the application of the affordances framework. At the end of stage 6, saturation was achieved.

Data were managed with NVIVO [33] software and written notes. Constructivist qualitative research assumes that themes do not emerge from the data but are constructed as part of a reflexive analytic processes [34]. Therefore, themes reported here should be considered as constructed. To improve rigour, themes were discussed in supervision where the aim was to raise potentially different interpretations. During the thematic analysis, SA kept reflective memos for each participant interview which detailed the development of the final analysis. Trial staff (the authors on this paper – including peer support workers, trial managers, clinical triage and research assistants) commented on whether they felt themes were an appropriate fit and the results represent consensus. The thematic analysis was conducted before participant access to the EMPOWER intervention was shut off. At this stage, participants could be described in terms of their app usage.

### Theoretical framework and reflexivity

We wanted to develop a deep understanding of how participants experienced using EMPOWER in daily life with a particular focus on identifying processes relevant for implementation. This research was conducted in a critical realist paradigm, which is a philosophy of science which assumes that there is a true social reality, but that we can only attempt to know it imperfectly by asking people about their perceptions [35]. Philosophical positions in process evaluation seem rarely reported which is why this has been shared explicitly by the authors, but the authors wish to be clear that they are describing a philosophical approach to the work of this process evaluation and are not proposing using critical realism as a method. For a summary of longstanding debates on critical realism as a method versus critical realism as a philosophy please see [36]. As critical realist philosophy assumes we can only know reality imperfectly, it is important to acknowledge the role of the researcher by considering reflexivity. Reflexivity is important in research [37], but typical reflexivity sections have been critiqued as only providing a shopping list of identities may give little insight into what brings a researcher to conduct research [38].

SA, who led on the analysis is a PhD student interested in understanding the implementation of EMPOWER with a particular focus on foregrounding end-user experiences. This has come from the recognition that testimonial injustice [39] is commonly enacted against people diagnosed with schizophrenia and their supporters (including mental health staff) which can mean their views are understood as “low quality” and “high risk of bias” within the technocratic hierarchy of evidence.

Reporting follows guidelines for qualitative research (Consolidated criteria for reporting qualitative research (COREQ)) [40] and a checklist for reporting can be seen in the appendix.

## Results

### Participants

In total, 16 patients (38% of people randomised to receive EMPOWER), 6 mental health staff (all psychiatric nurses – 27% of staff responsible for EMPOWER participants), and one carer (14%) completed one-on-one qualitative interviews. Interviews lasted from 11 min to an hour. To protect anonymity given the small sample, demographic details are limited and are show. Differences between interviewed patients and the rest of the randomised EMPOWER sample are in Table 1. The single carer participant did not consent to quotes being used, so SA presents reflections from that interview and has withheld all demographic details. Three further patient participants and one carer who were approached declined participation. Participants randomised to receive EMPOWER inputted data into the app ranging from 0 to 323 days mean 132.3 (SD = 111.10). Taking a mean split, we interviewed two patients who could be classified as “low users” (less than 132 days input) and 14 who could be classified as “high users” (more than 132 days input).

**Table 1:** Demographic comparison of interviewed and main trial participants

	Process Evaluation Interview Sample (n = 16)	Participants not interviewed (n = 26)	P value for test statistic
Age	47.2 (SD = 11.3)	39.5 (SD = 13.3)	0.05
Gender	56.25% female	46.1% female	0.75
Trial Site (UK or Australia)	75% UK	69.2% UK	0.95
Duration of contact with mental health services (months)	164.4 (SD = 124) (two missing values)	148.1 (SD = 122) (two missing values)	0.69
PANSS*Positive subscale	14.6 (SD = 5.2) Range = 9–27	15.0 (SD = 6.4) Range = 6* − 28 *minimum score	0.8
PANSS Negative Subscale	11.8 (SD = 3.4) Range = 7–18	15.2 (SD = 6.0) Range 8–30	0.02
Mean days inputting self-monitoring data into app per participant	218.56 (SD = 76.7)	79.23 (SD = 95.0)	< 0.001
Mean number of peer support worker contacts per participant	21.75 (SD = 7.5)	11.50 (SD = 7.7)	< 0.001

*Positive and Negative Syndrome Scale [41] with Van Der Gaag [42] factor structure.

### Overview of implementation themes

**Fig. 1:**
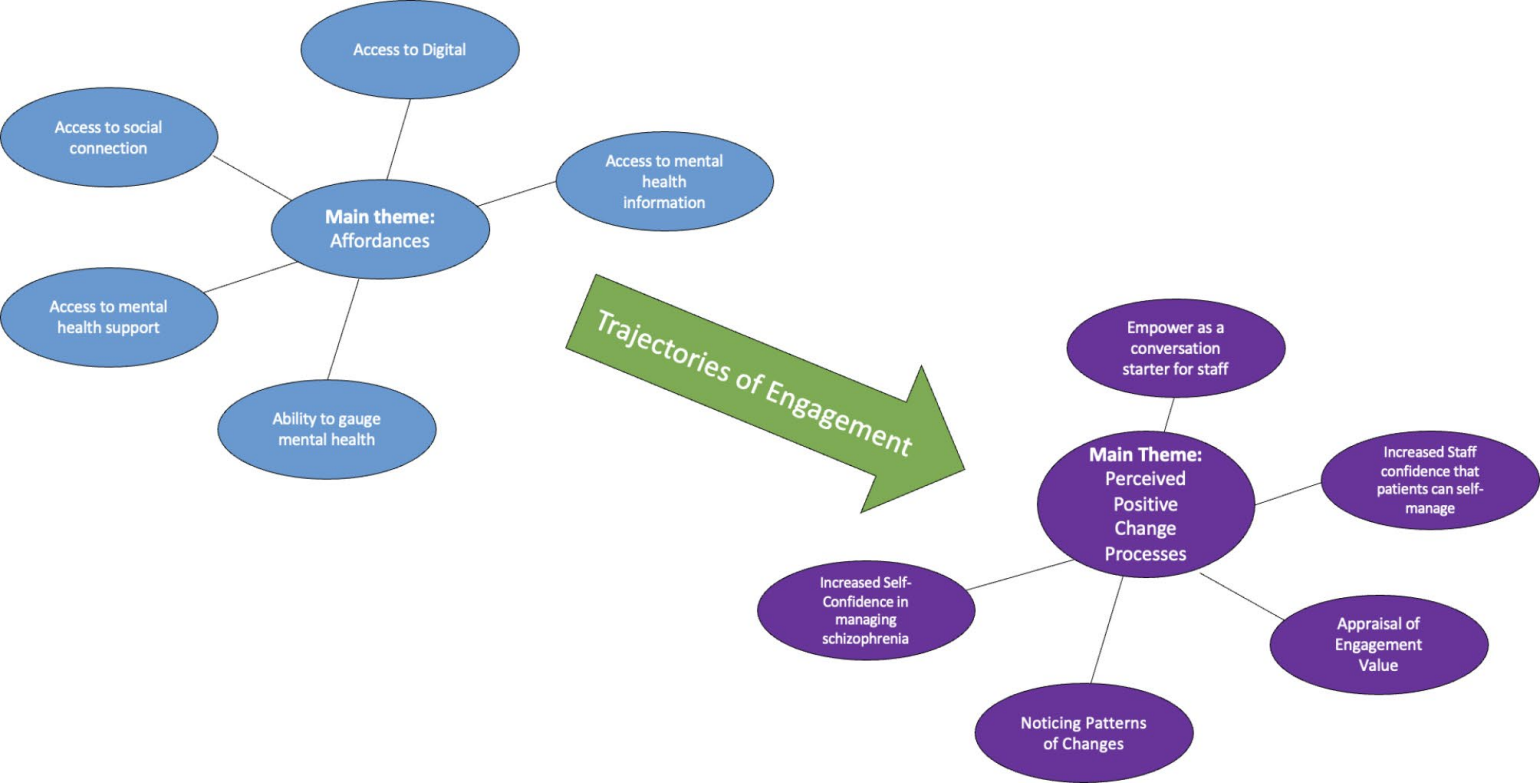
Shows a diagram summarising the relationship between the main themes of Affordances and Perceived Positive Change themes and the subthemes

From the qualitative analysis two overarching themes were constructed that were germane to understanding implementation behaviours within the EMPOWER trial which were Affordances and Perceived Positive Change Processes. Affordances were the engine house of implementation behaviours within the EMPOWER intervention. When applied to interventions like EMPOWER, this describes the processes underpinning how and why participants interacted with the various components of the intervention. Affordances spanned all EMPOWER components including self-monitoring, peer support workers, clinical triaging, wellbeing messages and diary function. The overarching affordances theme compromised of five subthemes which were: Access to Social Connection, Access to Digital, Access to Mental Health Support, the Ability to Gauge Mental Health and Access to Mental Health Information. The affordances framework proposed helped explain the multitude of implementation experiences featured within the qualitative interviews. Affordances helped circle the complex relationships between intervention capabilities and envisioned usage by participants. Affordances could be present at initial contact or developed over sustained engagement and act as a springboard for perceived positive change processes which appeared to explain continued implementation behaviours. These can be seen in Fig. 1.

While the affordances subthemes appeared to describe initial implementation experiences, the theme of Perceived Positive Change Processes describes the impact EMPOWER had upon participants from their subjective point of view which described what underpinned sustained implementation behaviours. Four subthemes were constructed which were: Increased Self-Confidence that Patients could Self-manage, Noticing Patterns and Changes, Using EMPOWER as a conversation starter and Appraising Engagement Value.

Because qualitative research can yield important insights into adaptations to intervention design, which may be necessary to improve participants’ experiences, we asked participants for suggestions, and their ideas for improvement which are also summarised.

## Affordances of EMPOWER

### Affording access to social connection

Many participants positively reflected upon their perception that EMPOWER afforded them access and the opportunity to have social connections with other people, this was typically expressed when discussing interactions with the peer support workers on the trial.

*“I’m happy someone [peer support worker] phoned me and saying my problems, listening to my problems, and talking together”* (Alesha, UK, 283 days app usage).

Moreover, the app itself was perceived as providing a sort of social connection even if people were not up for talking to a person directly. This seemed to be an important affordance and suggests the intervention could work for people with a range of communication styles who were driven by a need for connection.

*“I live on my own, I don’t see people. Apart from if I go out to the shops. I find that the app helps.… I like being asked how I’m doing every day. It’s because of the illness I’ve got, I can’t concentrate around other people. I go into a shell”* (Alesha, UK, 283 days app usage).

### Affording access to digital

For participants who had already have a smartphone it was clear that EMPOWER was perceived as affording access to the digital world which could be a very new experience. Quite beyond EMPOWER components, which the interview schedule was designed to explore, it was clear throughout the interviews that there had been an unexpected consequence of using the intervention. This was a clear example where an affordance emerged throughout a participant’s engagement in the trial and was best constructed as a process of discovery. In the example below, a participant described how the smartphone they had borrowed also meant they could now access things such as Google alongside the intervention.

*“Using Google and all these kinds of things, looking at websites and… weather… it’s opened up a whole load of new things”* (Matilda, UK, 281 days app usage).

Mental health staff also perceived that EMPOWER afforded access to the opportunity to develop skills and confidence in using technology even among people who are digitally excluded. In the example below, a staff member reflects surprise that the participant responded so well to using a digital device.

*“I couldn’t have been more wrong. [named patient] is probably the person you would think – out of all the people who attend this service, would be the least likely to use a mobile phone app. You know, I mean she has [not] got a mobile phone … it surprises me that she can even use that, you know.* (Gary, UK)

However, the digital nature of EMPOWER could be an implementation barrier depending on the context of a participant’s life. For example:

*“I’m off work just now it’s all right, but before it goes off when I’m at work. So, I’m either having to put it off till I get home, sometimes you miss it, or doing it in work, which is quite a private thing, you don’t really want to be doing it in your work”* (Keith, UK, 116 days app usage).

### Affording access to mental health support

EMPOWER clinical triage meant that if a change in a participant’s data suggested possible relapse, a clinical member of the EMPOWER team would check in with them. In practice, this meant that patients had access to clinicians beyond the scope of their standard mental health care. When considering implementation, this could be an especially important facilitator because it meant participants could access timely support. This is exemplified below where a participant shares how it was helpful to check out a negative change in their wellbeing in a timely manner rather than waiting to see their usual clinician.

*“We tried to get my mental health nurse. Couldn’t get them. They weren’t working Monday or Friday so it would be the following Tuesday before I would be able to get them. So, we phoned up [named clinician who provided clinical triage to trial participants] and spoke to them.”* (Matilda, UK, 281 days app usage).

However, the affordance of access to mental health support was dependent on how positive patients perceived mental health support to be. A participant who reported more difficult experiences with mental health services reflected that EMPOWER affording access to clinicians was aversive and acted as an implementation barrier. This participant had particularly low levels of engagement with the intervention, and this seemed to be explained by the triage process and speedier access to care not being perceived as helpful.

*“I don’t want people to cause a fuss over me…. I don’t need people phoning me upall the time and pestering me”* (Jay, UK, 21 days app usage).

The diary function was designed for self-reflection which means the data was not accessible by clinicians and therefore did not result in a response from them. It followed that if a participant was motivated to engage with EMPOWER because it afforded access to mental health support then they would not be likely to want to engage with components which did not have this feature. Indeed, this was suggested to be the case and the quote below highlights the importance of understanding affordances.

*“But nobody seen them. CPN, nobody seen them… So, no need to fill up the diary”* (Darius, UK, 219 days app usage).

### Affording a means to gauge mental health

Beyond providing access to mental health support, the EMPOWER intervention afforded a means to gauge mental health in a more general way through components such as the charts and the diary.

*“It’s quite good actually. I’ve done, I think I’m up to forty days in a row now. And that was what was good with EMPOWER especially when I was studying, and I wasn’t working. I would say a little diary entry each day just to sort of see how I was, and that was helpful too”* (Leonie, Australia, 271 days app usage).

However, it should be noted that while EMPOWER afforded an opportunity for gauging mental health – trial staff such as the peer support workers had an essential role as data interpreters and were a key reason for why participants understood EMPOWER as a means to gauge mental health. In the example below, a participant highlights the key role of the peer support worker in placing the participant self-monitoring data in a format the participant could understand.

*“I never used to understand charts, how they work. It’s not my type of thing. So, it’s hard to understand charts, for me. So [named peer support worker] was very helpful in explaining what it’s showing and what the projection is and the decline, and what it’s showing really, what’s happening”* (Sandy, Australia, 287 days app usage).

However, in this contrasting example a participant who stated they were not interested in gauging their own mental health and rather saw that task as something that should be done by a clinician describes how they were not interested in this affordance for themselves and therefore would not engage in looking at the charts, they nonetheless believed it could afford mental health staff an ability to gauge how they are.

*“I wouldn’t use the charts, really, you know what I mean? That would be something* for my nurse to see or something, or my doctor” (Alexander, UK, 265 days app usage).

### Affording access to mental health information

EMPOWER was seen as a source of potentially helpful mental health information, this could come from either accessing the wellbeing messages or through conversations with peer support workers.

*“[named peer support worker] talked to me about like mindfulness and meditationand stuff like that, and that’s something I’ve been looking into. So, I did find that quite useful. And talking to me about like, you can go and do group things and stuff. I don’t know how I’ll follow that yet, but he did suggest it to me. So, there are good things [the peer support worker] brings to the table”* (Keith, UK, 116 days app usage).

### Perceived positive change processes

Affordances provided the engagement “hook” which made engagement possible. Change was embodied through interaction with the EMPOWER intervention and described the positive impact of EMPOWER which participants reporting perceiving.

### Noticing patterns and changes

If EMPOWER afforded an opportunity to gauge mental health through self-monitoring, diary keeping and conversations with peer support workers, this could start a perceived positive change process of patients noticing patterns in their own wellbeing. Participants made explicit links between the intervention and noticing patterns and changes in their own wellbeing. Some participants appeared to have utilised the intervention to increase awareness about dynamic changes within their own mental health and sometimes reached profound realisations. In this example, a participant described how using the intervention helped her to notice how changes in her mental health were linked in with her menstrual cycle.

*“It was even good the graphs when it was, you know, female time of month or anything I might be feeling a bit crappier. And just being able to look at the graphs and go ’well actually, that’s why I was feeling a bit crappy’”* (Leonie, Australia, 271 days app usage).

*“I mean, the bad days, they showed me how I am. So yeah, literally bad days, you know what I mean? I’m back on my feet, what was better in the day before that. I look at the charts and see how they go, see the difference and say what do I do different”* (John, Australia, 141 days app usage ).

The perceived positive change process of noticing patterns and understanding change in wellbeing was not just limited to patients on their own – but also extended to whom they shared these insights with. This process can be seen in this excerpt where a participant reflected how the charts not only enabled them to see when they were “slipping” but this individual noticing became a joint understanding of patient wellbeing. This person’s process is demonstrated in this excerpt where a participant reflected how the charts help them see that they are becoming unwell but then help mental health staff understand also.

*“It’s just, I think the charts help me see where I’ve been slipping. That’s all I would*.

*say about that, just, yeah, the charts would help me notice when I’m slipping. And they help my CPN notice as well, yeah, when I was slipping down the charts as well”* (Agatha, UK, 138 days app usage).

### Change in appraisals of relapse

The programme theory was underpinned by an assumption EMPOWER would create change by reducing participants’ worries about having a relapse. While it is important to remain mindful that these qualitative interviews represent a cross-sectional snapshot of participant experiences, nonetheless a major theme was that fear of relapse varied, and while some participants seemed to report feeling less concerned about relapse, this varied.

*“Sometimes I do [worry about relapse], but I sort of think about it and go “well, it might never happen again.” Like you I’m stable, I’ve got good accommodation, I’ve got a good job, things are okay. So, I accept that it could happen in the future, but it’s not something I think about.”* (Leonie, Australia, 271 days app usage).

Relapse could even become a more frightening prospect when people were further along a recovery journey and the potential consequences and losses arising from a relapse increased.

*“I dread it more. …how far I’ve come, I think, to relapse now would just be a crying shame.”* (Michaela, UK, 195 days app usage).

### Differing experiences of implementation underpinned by perceived positive changes processes

Affordances shaped intervention implementation. For example, participants demonstrated different implementation journeys which were linked to their appraisal of need for continued use of EMPOWER.

When speaking with patient participants, it seemed that sustained implementation behaviours were underpinned by whether the participant perceived value in continuing to implement the intervention. Perceiving a positive value was an implementation facilitator, whereas no longer perceiving a positive value or never having had perceived a positive value acted as an implementation barrier. Taken further, this suggests engagement with EMPOWER is best understood as an interactional process determined by participants balancing the value of continuing. For example, here a participant states that “I’m a bit over it now” as they no longer feel they are gaining positive benefit from being involved and continuing to use EMPOWER continuously does not make sense.

For want of a better phrase I’m a bit over it now. I think it was really, really good to start with, but I think as I’ve gotten more well and as I’ve got back to the workforce and things like that, I haven’t needed as much support…. I think in the long term, once patients became more stable, I think the need for it decreases.

(Leonie, Australia, 271 days app usage)

In further support of this, there were examples of participants who reported that their appraisal of engagement value was still on a positive trajectory, and they had not yet experienced the perceived positive change process described in the account above which was leaning towards a termination of engagement:

*“One thing that I have worried about is when this finishes, I’ll miss it and I hope I’ll continue to reflect each day, to invest a bit of time in myself, how I’ve been feeling, what the day’s been like, or what the week’s been like.”* (Matilda, UK, 281 days app usage).

Self-reporting mental health data every day could be tedious for participants, however if participants still perceived a positive benefit from engagement, it seemed that this made them motivated to keep going and continue to implement the intervention.

*“Just force of habit, you know. Just like taking medication…. It’s a bit tedious, you know. Sometimes you can’t be bothered going through it all because it’s the same every day, you know. I can do it quite quickly now, so I can get the answers up quite quickly, you know.”* (Nancy, UK, 261 days app usage).

### Increased self-confidence in managing Schizophrenia

When speaking to some patient participants, it was clear that some participants appeared to have developed confidence that they could self-management their condition a bit better through their experiences of implementing EMPOWER.

*“[EMPOWER] means that I’m not hiding away from my illness and I’m not ignoring itand I’m not pretending it’s not happening and carrying on regardless … since I’ve started using the App it’s made that pathway to recovery much quicker.”* (Emily, UK, 282 days app usage).

But this varied, here a participant states that while they feel they have improved – it is not at all attributed to using the intervention.

*SA: “So, since you’ve started using EMPOWER, have you noticed any*

*Changes in how you manage your own wellbeing?”*

*Participant: “I’m getting better. I’ve been getting better for the last year but it’s not your fault, it’s my medicine’s fault.”* (Seumas, UK, 261 days app usage).

## Staff

### EMPOWER as a conversation starter

A perceived positive change process for mental health staff which encouraged them to support implementation was EMPOWER functioning as a conversation starter. The main trial findings demonstrated that data sharing between patients and mental health staff was not routine. For staff, a key change process was that staff used the fact the participant was part of the EMPOWER study and self-monitoring their mental health to open conversations about wellbeing – rather than looking at charts:

*“So, generally I guess I use the app just to start discussions around… you know, howshe is feeling really.”* (Edith (Staff) UK).

*“I sort of shy away from using the data in a sense, I really want to keep it - I didn’t want [named patient] to feel I was looking at her data and making a judgement… I tended to ask her how she was going rather than “I looked at your data and thought… as it takes it away from a personable experience.” (Philippa (Staff), Australia)*.

### Increased staff confidence that patients can Self-manage

Beyond using EMPOWER participation as an opportunity to open up conversations about patient mental health status, across all six staff interviews it was constructed that the very fact patients were engaged in the trial meant staff were afforded confidence patients could self-manage to a degree and they could simply let the participant get on with it. This perceived positive change process is exemplified by member of the community mental health team reflecting their trust in the patient engaging with EMPOWER had engendered staff confidence in the ability of patients to self-manage.

*“But knowing that the app’s there, knowing that she’s responding to that… knowing that she’s the support from EMPOWER itself, getting phone calls from the Peer Support workers - the nurse, it’s made me more confident in her ability to do it.”* (Gary (Staff), UK).

### Improvement suggestions

The EMPOWER trial tested whether the intervention was feasible, this means qualitative interviews had merit in gathering end user suggestions for intervention refinement which may increase the facilitation of implementation behaviours. These are not ‘themes’ so are expressed as a list with evidence from the qualitative interviews for transparency.

### A phone with better battery life

People who were given a smartphone to use stated that the phone did not have good battery life.

*“The battery life’s not that great. The battery life isn’t that great at all. I’m charging it everynight for an hour or two”* (Michaela, 195 days app usage, UK).

### Being provided with a phone cover

Participants were given a phone without a phone cover. Due to the fragility of the smartphone some participants were concerned about breaking the phone if they took it outside or dropped it. Offering a smartphone case might make using the app more feasible.

SA: “I know you mentioned that you were worried about dropping the phone would the team providing you with a phone case to make it sturdy would that be helpful?”

Participant : “Yeah that would be helpful yeah uh-huh.” *(Emily, UK, 282 days app usage)*.

### Being able to choose questionnaire timing

The pseudorandomised prompt timing was a key frustration along with the limited time available to answer it. Participants suggested it would be good to change the timing and bring this under participant control.

*“To be honest with you, I prefer to pick a time of my own. And I know that at that time that I sit down and devote attention that I want to.”* (Matilda, UK, 281 days app usage).

### Not asking questions (or sending wellbeing messages to) participants about experiences participants do not have

This suggestion was particularly marked from people who did not hear voices. Participants would appreciate an intervention which is more tailored towards their own mental health rather than assumptions about what they are likely to experience by virtue of having a particular psychiatric label.

*“I’ve found a few of them [wellbeing messages] helpful but mostly they’re all about voices, and I don’t really get voices, so they’re not applicable to me, most of them. So, I think they could be more tailored to the answers you’ve put.”* (Keith, UK, 116 days app usage).

### Being able to save message content in a personal bank

The EMPOWER messages that gave participant information on managing psychosis, quotes and links to videos were refreshed daily which meant participants could not save ones that were meaningful to them. Several participants remarked that this would be improved by enabling them to save messages.

*“[the messages] disappear the next day so they’re not there. And I’ll think “oh, maybe I could listen to that one again”, and it’s not there anymore. So, if it was somewhere where you could click on it the next day or however long you wanted to keep track of it, that would be good.”* (Agatha, UK, 138 days app usage).

## Discussion

This study utilised a thematic analysis approach to construct themes relevant for understanding implementation behaviours within the EMPOWER feasibility trial from the perspectives of patients, mental health staff and one carer. From the results of the analysis, two key themes were identified that appear relevant to understand the mechanisms underpinning the relatively high implementation behaviour observed within the EMPOWER trial and will likely be of interest to researchers looking to develop digital interventions for people who experience psychosis, and in particular, those who have recently experienced a relapse.

EMPOWER was a complex intervention which blended multiple components. All three key main EMPOWER components (clinical triage, self-monitoring and self-management with peer support worker support) appeared to be important and were appraised positively by patients, mental health staff and the one carer we spoke with. While some participants found the intervention difficult to use at first, patient-participants generally expressed confidence in using the mobile phone-based intervention components. This is noteworthy because some had never used a smartphone before, and there was concern expressed before the EMPOWER trial that older and digitally excluded people may struggle to use an app-based intervention [28]. In general, all intervention components were perceived as being simple to use with the support from trial staff in supporting engagement being viewed positively.

Initially, implementation by patient participants appeared driven by what the intervention afforded them (such as social connection). However, sustained implementation appeared driven by patients perceiving that the app was creating positive change in their life such as increased confidence in self-managing. The themes constructed in this analysis helped develop a theoretical account for why these implementation behaviours occurred. The first key concept was affordances. EMPOWER affordances can be described as offerings which may or may not be in line with how participants (patients, staff, and carers) envision how and why they will interact with the intervention [43]. The high frequency of implementation behaviours in the EMPOWER trial appeared best explained by the intervention offering a range of potential affordances that were personally meaningful to participants. For example, participants who were isolated were afforded human contact, in contrast, participants who did not want to speak to people were also afforded the opportunity to communicate how they were feeling via self-monitoring without talking to another person. Due to the flexibility of EMPOWER, both distinct affordances could be satisfied resulting in motivation to implement the intervention.

Patients reported that taking part in the intervention afforded them increased access to information about psychosis generally through conversations with peer workers or wellbeing messages as well as direct support from clinical trial staff during crisis events. Previous research has indicated that people diagnosed with schizophrenia perceive that digital interventions can afford them another source of support [12, 13, 44]. Expanding upon these previous findings, this research suggests mental health staff believe digital interventions can afford increased access to information and support for their patients, with this being generally being perceived positively and leading to staff taking a more hands-off approach. “Face-to-face” components of blended interventions has been an implementation facilitator noted in other qualitative research with people who experience psychosis [45] and bipolar disorder [46]. The current analysis builds on this and suggests mental health staff also viewed the increased human contact available to people they support in a positive light. Affordances also presented a useful theoretical framework for understanding cases where users did not implement the EMPOWER intervention. For example, a participant with very low levels of intervention usage who had experienced difficulties with mental health services reported that the intervention afforded access to a mental health professional contacting them during triage which was perceived negatively and acted as an implementation barrier. Another patient participant with low usage expressed frustration that the intervention assessed voice hearing which was not something they experienced which acted as an implementation barrier because the increased access to mental health information which was valued in high users was not appropriate for them. Tailoring of digital interventions has been noted to increase motivation to implement digital interventions because information is more relevant for the person [47] and offering tailoring may increase likelihood of access to information about mental health being afforded to more patients.

While affordances helped explain early implementation, sustained implementation appeared driven by patients, carers and mental health staff perceiving positive change. This was distinguished from affordances because these themes described experiences beyond EMPOWER offering people something but rather extended to people noticing a positive change within their daily lives. If participants noticed a positive change from using EMPOWER – they continued to do so. However, participants who had stopped noticing positive change or who never found the intervention helpful dropped off. This “perceived positive change processes” theme is very similar to the theme of perceived effectiveness noted by Steare and colleagues in their qualitative evaluation of a digital self-management psychosis app [48]. The findings here indicate that patient participants continued to engage in implementation behaviours if they perceived EMPOWER was creating positive change. This supports a call for researchers to further understand engagement in relation to the purpose of digital interventions [49] and suggests affordances and perceived intervention value may be key for achieving this.

EMPOWER was designed with an assumption that patients would share data with mental health staff who support them to reduce inherent uncertainties within EWS based relapse prevention. While there were rare examples in the interviews of patients sharing data with mental health staff to develop a shared understanding of wellbeing, typically mental health staff opted to use the fact the patient was part of the study to open conversations rather than relying on data to understand how a patient was doing. This appeared to be a positive change process for mental health staff. Staff not feeling comfortable using data generated by digital interventions is a common implementation barrier [50] and staff discomfort may explain the low levels of data sharing between patients and staff observed in the main trial [11]. However, rather than acting as an implementation barrier, staff appeared to come to believe that patients who were not in crisis that were using EMPOWER could self-manage which may have led to staff taking an even more hands-off approach to data sharing. This seemed to act as an implementation facilitator because staff kept encouraging patients to keep implementing the intervention.

### Limitations

These findings should be considered in light of limitations. The Interviews were conducted with a small sub-sample of end-users and seven patients dropped out of the trial before process evaluation interviews began and withdrew consent for future data collection, which means the perspectives of people who used the intervention less because they dropped out are missing from this study. Therefore, the results likely present factors which are more relevant to patients who had more positive experiences and people we spoke to had engaged significantly more with both self-monitoring and peer support. Future research would be greatly enhanced by discovering why the intervention was less suitable for some people than others, and by conducting more interviews with people who drop out of studies early or decline to take part in a digital intervention trial in the first place. Furthermore, since all interviewees were already participants in a trial, their views may not be representative of how EMPOWER would be used within routine mental health care settings. Only one carer participated. Carers reported feeling relatively uninvolved within routine relapse management [10] and it may be the case that low carer participation within EMPOWER reflects this. Carer views on taking part in dyadic research are much needed.

Going forward, patient public involvement work with carers within future process evaluation research (especially exploring best recruitment practices) may be of merit here. The Interview schedules were created to understand implementation behaviours by mapping closely to the process evaluation framework [21], which may have limited the quality and breadth of the qualitative data. Additionally, member checking (where participants comment on the accuracy of the analysis) would likely have enhanced the research and minimised the risk of researchers misunderstanding participant views [51].

### Implications

The EMPOWER trial itself had pre-defined criteria for determining intervention acceptability and feasibility [11] and positive results suggest the intervention has the potential to be well implemented. Our findings from a qualitative process evaluation focused on understanding implementation behaviour suggest ideas for improvement which may improve implementation in subsequent intervention iterations. This is important because intervention participants have valuable insight into what an implementable intervention means for them [52]. For example, participants indicated they would like to be able to self-initiate self-monitoring rather than relying on the existing random schedule of EMPOWER because this would make it easier to implement EMPOWER into the context of their daily lives. While evidence from the ACTISSIST self-monitoring intervention indicates engagement tends to be higher from app initiated prompting rather than self-initiated self-monitoring [53] it appears pertinent to recommend future iterations still offer a self-initiated option.

As identified in previous qualitative research with people who experience psychosis [48, 54], participants spontaneously identified potential solutions to problems they encountered with digital interventions that would make them easier to implement, such as providing a phone cover or a phone with a better battery life. These are simple yet powerful suggestions which may help develop digital interventions which can be more easily implemented by patients.

EMPOWER patient participants had all had a recent relapse. Several participants who were still using EMPOWER at the point of interview remarked that they were no longer noticing any positive changes from using the intervention and felt that it had offered them all it could, and they felt they had moved on in their recovery journey. Since mental health staff did not appear to be actively involved in implementing the intervention beyond providing encouragement and curiosity, there is a need to focus on why patients implemented EMPOWER into the context of their daily life. These findings indicate that there is likely to be an optimal engagement period, but that this will likely very much depend on the individual patient.

Going forward, a potential role for peer support workers, whom many patient participants endorsed a trusting relationship with, could be to have discussions with people whom they support about the pros and cons of continuous usage so that patients can make an informed choice about whether continuous monitoring tools are benefifical in their recovery.

Interventions which are flexible and empower participants to discover affordances which are personally meaningful to them seem likely to be well implemented. Affordances are an interaction between a person and an object in their environment, with this in mind one key recommendation from our research is that future interventions work closely with patients to discover what would drive them to engage. Co-design and user engagement is in line with current guidance from the MRC complex intervention development framework [24].

## Conclusion

This study reported what underpinned implementation behaviours from the point of view of patients, mental health staff and one carer when interacting with EMPOWER in daily life.

When speaking with patients, initial implementation appeared best explained as EMPOWER offering a range of affordances which could act as implementation barriers or facilitators depending on individual needs and wants. However, ongoing implementation behaviours were better explained by patients and staff perceiving that the intervention had created positive change for them which were having a beneficial impact upon everyday life. Sustained engagement seemed dependent on patients continuing to perceive positive value from the intervention which suggests there may be optimal engagement periods. These findings contribute to our understanding of what underpins relatively successful implementation of digital self-management interventions for psychosis and highlight key suggestions for how to improve interventions further.

### Electronic supplementary material

Below is the link to the electronic supplementary material.

Supplementary Material 1: Consolidated criteria for reporting qualitative studies (COREQ): 32-item checklist.

## Data Availability

The datasets analysed during the current study available from the chief investigator (AG) on reasonable request.
